# Data supporting assessment for nitrous oxide emissions from soils under traditional cropland and apple orchard in the Loess Plateau of China

**DOI:** 10.1016/j.dib.2018.10.151

**Published:** 2018-11-01

**Authors:** Junzhu Pang, Xiaoke Wang, Changhui Peng, Yujing Mu, Zhiyun Ouyang, Fei Lu, Hongxing Zhang, Shuoxin Zhang, Wenzhao Liu

**Affiliations:** a Laboratory for Ecological Forecasting and Global Change, College of Forestry, Northwest A&F University, Yangling 712100, China; b Research Center for Eco-Environmental Sciences, Chinese -Academy of Sciences, Beijing 100085, China; c Collage of Forestry, Northwest A&F University, Yangling 712100, China; d Department of Atmospheric Chemistry and Air Pollution Control Technology, Research Center for Eco-environmental Sciences, Chinese Academy of Sciences, Beijing 100085; e Institute of Soil and Water Conservation, Chinese Academy of Sciences, Yangling 712100, China

## Abstract

The data presented in this article relates to the research article entitled “Nitrous oxide emissions from soils under traditional cropland and apple orchard in the semi-arid Loess Plateau of China” (https://doi.org/10.1016/j.dib.2016.08.027) (Pang et al., 2019). The dataset includes soil N_2_O emissions for two land use types (wheat field and apple orchard) in the semi-arid Loess Plateau and related environmental factors, such as soil temperature and soil moisture. In addition, the estimated annual average and seasonal cumulative emissions of N_2_O are presented here. Nitrous oxide emissions were measured by static, closed chamber methods. The data provides evidence for the difference in N_2_O emissions among two dominant land uses on the Loess Plateau of China.

**Specifications table**

**Table:** 

Subject area	*Ecology, environmental sciences.*
More specific subject area	*Nitrous oxide emissions.*
Type of data	*Table.*
How data was acquired	*Filed measurement, sampling and laboratory analysis.*
Data format	*Raw and analyzed.*
Experimental factors	*Nitrous oxide emissions from crop land and orchard.*
Experimental features	*A rain-fed winter wheat field and an apple orchard that was converted from the wheat field in 1984.*
Data source location	*107°41′E, 35°14′N, Changwu country, Shaanxi Province, China.*
Data accessibility	*Data is with this article and related reference.*
Related research article	*Pang et al., Nitrous oxide emissions from soils under traditional cropland and apple orchard in the semi-arid Loess Plateau of China. Agric. Ecosyst. Environ. 2019, 269(1):116–124.*https://doi.org/10.1016/j.dib.2016.08.027*[1]*.

**Value of the data**

• This data contains key information for N_2_O emissions from traditional cropland and apple orchard soils in China׳s Loess Plateau.
• This data can be used to estimate N_2_O emission from agricultural soil in temperate semi-arid region of China.
• This data can be used to estimate the impacts of land use change and the seasonal and annual fluctuation of precipitation on N_2_O emissions on the Loess Plateau in China.

## 1 Data

This article presents soil temperature (°C, 10 cm), soil water-filled pore space (WFPS) (%, 0–30 cm) and N_2_O emission data for two land use types (wheat fields and apple orchards) on the south central Loess Plateau in China (Table 1, Table 2, Table 3, Table 4). Table 5 includes the estimates of annual average and seasonal cumulative emissions of N_2_O.

**Table 1:** Soil temperature, WFPS and N_2_O emissions of the wheat field.

Date	T (°C)	WFPS (%)	N_2_O emissions (µg N_2_O m^−2^ h^−^^1^)
2007-4-11	13.3	21.25	38.78
2007-4-29	13.5	16.54	21.31
2007-5-7	20.4	12.49	20.22
2007-5-12	15.7	21.4	21.66
2007-5-14	17.2	19.05	14.45
2007-5-17	16.5	14.58	19.36
2007-5-24	16.7	18.34	28.06
2007-6-11	20.3	42.58	28.26
2007-6-23	19.7	46.3	19.38
2007-6-25	21.7	40.22	10.16
2007-7-10	20.3	42.41	21.9
2007-7-26	19.5	32.36	42.54
2007-7-29	21.1	43.66	56.08
2007-8-1	21.2	37.34	23.18
2007-8-3	19.2	35.48	26.4
2007-8-14	21.5	21.59	24.79
2007-9-2	22.2	28.86	17.02
2007-9-18	17.6	31.85	41.62
2007-9-27	15.3	42.89	29.98
2007-10-4	14.4	43.12	65.04
2007-10-5	15.3	43.34	93.4
2007-10-10	9.5	43.57	23.06
2007-10-12	9	43.92	23.16
2007-10-13	9.7	48.11	32.38
2007-10-14	8.7	52.24	32.38
2007-10-16	7.9	52.83	19.78
2007-10-18	12.1	42.53	17.88
2007-10-21	8.5	49.46	14.38
2007-10-30	6	61.82	27.84
2007-11-17	3.5	52.28	21.52
2007-12-6	−1.4	44.22	7.94
2007-12-16	0	38.3	8.44
2008-1-3	−1.3	43.51	19.98
2008-2-23	0.2	76.07	16.94
2008-2-29	0.2	41.33	52.68
2008-3-7	1.4	42.16	8.7
2008-3-14	4.3	44.24	34.56
2008-3-24	3.6	40.45	15.38
2008-4-9	10.6	29.78	12.68
2008-4-25	8.1	33.13	18.2
2008-5-8	15.1	24.46	8.92
2008-5-23	18.4	18.98	12.08
2008-6-7	17.4	53.27	10.88
2008-6-8	15.4	42.89	34.86
2008-6-25	23.1	47.13	18.38
2008-7-3	21.4	50.6	38.04
2008-7-19	19.8	58.64	37.9
2008-8-3	24.2	36.48	52.64
2008-8-4	25	33.84	69.38
2008-8-5	24.8	36.48	18.48
2008-8-6	25	32.06	51.48
2008-8-7	26.8	30.51	46.9
2008-8-10	18.9	38.5	45.16
2008-8-25	16.9	36.34	44.74
2008-9-11	11.9	47.98	34.66
2008-9-14	16.4	42.53	50.37
2008-9-15	17.1	40.61	33.42
2008-9-16	15.7	38.74	74.72
2008-9-17	16.2	36.43	44.6
2008-9-18	16.7	35.7	62.54
2008-9-19	16.6	32.99	26.5
2008-9-20	17.3	32.86	13.02
2008-9-23	15.8	48.59	76.28
2008-9-30	10.4	49.28	43.86
2008-10-2	13.4	47.76	57.75
2008-10-4	15	41.28	15
2008-10-6	11.3	39.63	19.84
2008-10-18	10.4	30.35	12.78
2008-11-3	4	26.45	9.68
2008-11-18	1.6	28.29	18.42
2008-12-3	1.8	25.76	10.268
2008-12-18	−0.9	26.65	7.44
2009-1-5	−0.8	26.03	11.9
2009-1-22	−0.9	25.04	8.8
2009-2-10	−0.2	26.06	25.44
2009-2-28	0.9	30.78	2.58
2009-3-7	1.7	27.12	29
2009-3-17	0.9	28.12	29.7
2009-3-28	5.5	24.65	49.02

**Table 2:** Soil temperature, WFPS and N_2_O emissions of the D 2.5 site in the apple orchard.

Date	T (°C)	WFPS (%)	N_2_O emissions (µg N_2_O m^−^^2^ h^−^^1^)
2007-4-11	24	24.62	16.04
2007-4-29	28.3	22.79	31.88
2007-5-7	39.3	22.16	31.648
2007-5-12	24.5	24.69	38.56
2007-5-14	22.1	20.87	24.26
2007-5-17	25.1	21.69	30.44
2007-5-24	24.3	20.69	11.58
2007-6-11	24.1	41.32	26.68
2007-6-23	25.3	37.04	5.74
2007-6-25	23.8	38.44	19.88
2007-7-10	19.6	24.68	15.858
2007-7-26	18.6	27.25	8.06
2007-8-14	20.1	47.81	21.86
2007-9-2	22.1	45.93	32.7
2007-9-18	18	44.75	32.58
2007-10-14	9.9	46.83	14.34
2007-10-30	7.6	42.32	21.04
2007-11-17	4.8	36.46	13.48
2007-12-6	−0.03	48.6	16.34
2007-12-16	0.6	47.86	9.48
2008-1-3	–0.8	66.17	29.06
2008-2-23	0.2	41.63	75.16
2008-2-29	0.8	30.26	70.8
2008-3-7	2.4	42.27	14.98
2008-3-14	6.7	46.64	52.08
2008-3-24	7.6	40.3	22.84
2008-4-9	13.3	36.25	9.8
2008-4-25	12.9	36.88	25.74
2008-5-8	16.3	28.71	23.04
2008-5-23	22.3	22.96	26.88
2008-6-7	18.2	43.52	28.06
2008-6-8	18.7	26.63	73.46
2008-6-25	27.4	36.44	60.3
2008-7-3	22.8	42.61	58.34
2008-7-19	19.1	45.48	52.6
2008-8-10	21.2	44.78	33.56
2008-8-25	18.8	38.63	74.46
2008-9-11	14.1	49.05	32.68
2008-9-30	11.9	60.54	25.41
2008-10-18	11.2	30.75	22.32
2008-11-3	5.1	28.1	16.2
2008-11-18	1.7	28.48	10.6
2008-12-3	0.8	25.67	9.6
2008-12-18	0.6	25.79	7.38
2009-1-5	−0.8	25.1	11.56
2009-1-22	−0.9	25.39	6.08
2009-2-10	−0.2	25.19	11.46
2009-2-28	0.9	31.54	28.74
2009-3-7	5.5	27.77	12.64
2009-3-17	1.8	28.12	28.98
2009-3-28	8.5	23.38	33.74

**Table 3:** Soil temperature, WFPS and N_2_O emissions of the D 1.5 site in the apple orchard.

Date	T (°C)	WFPS (%)	N_2_O emissions (µg N_2_O m^−^^2^ h^−^^1^)
2007-4-11	23.5	25.45	28.22
2007-4-29	24.1	20.92	20.808
2007-5-7	33.4	22.1	29.3
2007-5-12	19.6	19.23	25.66
2007-5-14	21.2	17.68	24.758
2007-5-17	24.2	19.22	20.2
2007-5-24	21.7	23.64	19.92
2007-6-11	22	37.1	30.94
2007-6-23	23.9	32.4	15.76
2007-6-25	22.3	30.16	21.02
2007-7-10	20.7	21.45	28.48
2007-7-26	18.9	30.64	29.74
2007-8-14	20.1	36.65	5.28
2007-9-2	22.5	30.02	27.54
2007-9-18	20.8	46.27	32.28
2007-10-14	9.9	47.43	19.36
2007-10-30	7.6	40.47	13.74
2007-11-17	4.8	39.63	8.64
2007-12-6	−0.08	41.58	21.776
2007-12-16	1.1	47.29	17.154
2008-1-3	−1.2	58.69	21.96
2008-2-23	1.7	30.31	19.9
2008-2-29	1	31.48	69.66
2008-3-7	3.4	39.24	22.94
2008-3-14	8.4	34.17	29.54
2008-3-24	8.1	37.65	17.04
2008-3-29	6.3	37.12	23.72
2008-3-30	4.8	36.28	25.71
2008-3-31	7.1	38.14	38.64
2008-4-1	6.5	33.43	21.52
2008-4-2	6	33.18	25.48
2008-4-4	6.2	32.87	111.34
2008-4-6	9.5	32.72	12.18
2008-4-9	13.6	40.23	31.98
2008-4-12	8.4	34.28	21.98
2008-4-15	9.1	30.97	12.3
2008-4-25	12.9	27.7	40.16
2008-5-8	16.6	17.9	43.04
2008-5-23	20.4	65.33	3.34
2008-6-7	17.6	30.54	36.18
2008-6-8	17.3	37.51	28.56
2008-6-25	21.3	66.34	125.44
2008-7-3	21.8	48.34	91.16
2008-7-19	20	36.43	54.3
2008-8-10	19.6	51.16	87.4
2008-8-25	17.6	36.24	23.02
2008-9-11	13.7	48.4	48.1
2008-9-30	11.6	55.27	13.04
2008-10-18	12.5	31.58	31.74
2008-11-3	5.7	29.23	13.62
2008-11-18	1.5	30.39	7.4
2008-12-3	0.2	28.74	9.388
2008-12-18	0.1	29.01	10.48
2009-1-5	−0.8	23.83	16.88
2009-1-22	−0.9	27.07	7.02
2009-2-10	−0.2	25.85	11.46
2009-2-28	0.9	31.12	6.08
2009-3-7	5.2	27.73	31.24
2009-3-17	0.9	27.7	6.48
2009-3-20	9.6	26.16	95.39
2009-3-21	11.8	23.58	6.7
2009-3-22	5.5	21.5	14.054
2009-3-23	6.3	30.27	42.672
2009-3-24	6.6	18.28	2.482
2009-3-25	5.9	16.53	226
2009-3-26	6	18.27	7.942
2009-3-28	7.7	25.02	60.48
2009-3-30	5.6	25.36	32.596

**Table 4:** Soil temperature, WFPS and N_2_O emissions of the D 0.5 site in the apple orchard.

Date	T (°C)	WFPS (%)	N_2_O emissions (µg N_2_O m^−2^ h^−^^1^)
2007-4-11	22.9	25.33	20.16
2007-4-29	19.7	21.37	27.3
2007-5-7	28.7	20.16	23.64
2007-5-12	18.1	22.14	10.82
2007-5-14	20.2	19.23	13.794
2007-5-17	20	20.7	16.08
2007-5-24	18.9	20.98	13.72
2007-6-11	22.2	12.4	18.82
2007-6-23	20.4	34.94	19.82
2007-6-25	21.5	32.33	27.8
2007-6-27	21.4	21.54	26.33
2007-6-28	22.8	21.09	68.4
2007-6-29	22.5	20.47	70.835
2007-6-30	20.6	19.62	94.9
2007-7-1	17.3	19.07	61.62
2007-7-3	20.8	25.64	85.73
2007-7-10	19.8	33.27	23.82
2007-7-26	18.7	28.13	46.5
2007-8-14	19.2	33.5	19.02
2007-9-2	21.8	38.96	10.32
2007-9-18	19.5	32.74	72.54
2007-10-14	10.7	62.29	15.9
2007-10-30	7.6	49.87	13.94
2007-11-17	4.8	36.18	11.94
2007-12-6	−0.01	36.4	8.76
2007-12-16	0.4	33.74	9.58
2008-1-3	−0.1	37.96	14.94
2008-2-23	2.3	70.79	108.6
2008-2-29	1	32.99	37.34
2008-3-7	3.9	33.25	38.36
2008-3-14	8.3	36.77	21.7
2008-3-24	6.3	37.98	13.88
2008-4-9	12.6	36.22	31.78
2008-4-25	12.4	34.77	15.26
2008-5-8	15.9	29.92	27.1
2008-5-23	19.5	18.25	32.16
2008-6-7	17.6	42.05	29.3
2008-6-8	17.65	29.13	35.78
2008-6-25	20	39.7	67.04
2008-6-2	21.4	21.54	18.86
2008-6-28	22.8	21.09	63.24
2008-6-29	22.5	20.47	79.64
2008-6-30	20.6	23.34	105.12
2008-7-1	17.3	30.18	67.5
2008-7-3	20.8	41.51	130.26
2008-7-19	19.6	45.66	113.06
2008-8-10	19.4	48.28	63.92
2008-8-25	18.2	35.89	30.36
2008-9-11	13.9	46.09	50.54
2008-9-30	11.4	53.38	14.34
2008-10-18	13.6	30.35	13.82
2008-11-3	7.4	26.68	11.73
2008-11-18	1.3	28.59	6.56
2008-12-3	2.6	24.82	16.718
2008-12-18	−0.2	23	16.28
2009-1-5	−0.8	22.97	22.64
2009-1-22	−0.9	22.69	5.56
2009-2-10	−0.2	23.28	7.8
2009-2-28	0.9	30.21	3.32
2009-3-7	5.6	25.13	29.66
2009-3-17	1.7	25.54	15.68
2009-3-28	7.8	22.16	21.04

**Table 5:** Annual average and seasonal cumulative emissions of N_2_O (kg N_2_O h m^−^^2^ yr^−1^).

	Annual average N_2_O emission ± se (kg N_2_O h m^−^^2^ yr^−^^1^)	2007 Summer N_2_O emission ± se (kg N_2_O h m^−2^ yr^−1^)	2008 Summer N_2_O emission ± se (kg N_2_O h m^−^^2^ yr^−^^1^)	2008 Freeze -thaw N_2_O emission ± se (kg N_2_O h m^−^^2^ yr^−^^1^)	2009 Freeze -thaw N_2_O emission ± se (kg N_2_O h m^−^^2^ yr^−^^1^)
Wheat land	2.14 ± 0.08	0.56 ± 0.04	0.78 ± 0.11	0.21 ± 0.04	0.20 ± 0.06
Total apple orchard	2.40 ± 0.15	0.47 ± 0.12	1.31 ± 0.20	0.248 ± 0.03	0.17 ± 0.03

## 2 Experimental design, materials, and methods

### 2.1 Experimental design

Two neighboring fields on flat land were selected for N_2_O measurement: (i) a winter wheat field and (ii) an apple orchard that was converted from a wheat field in 1984. In the wheat field, three sub-plots were randomly selected and three chamber bases were installed in each sub-plot (nine chamber bases in total). In the apple orchard, three sub-plots were selected. At each sub-plot, three sites were selected at distances of 2.5 m, 1.5 m and 0.5 m away from tree rows (D 0.5, D 1.5 and D 2.5). Three chamber bases were installed in each sub-plot (nine chamber bases in total).

### 2.2 Material and methods

Nitrous oxide emissions were measured by static, closed chamber methods. Each chamber (50 cm × 50 cm × 50 cm) was constructed with an aluminum alloy frame having transparent PVC and was covered by adiabatic film. The N_2_O concentration was analyzed by gas chromatography (Model SP3410, Beijing Analytical Instrument Factory), fitted with an electron capture detector (ECD). During each sampling event, soil temperature (0–10 cm), and soil moisture (0–30 cm) were monitored simultaneously. Soil temperatures were measured with a SN2202 digital thermo detector (Sinan Instrument Plant of Beijing Normal University). Soil moisture (0–30 cm) was determined gravimetrically after oven-drying (at 105 °C for 24 h). During the study, soil water-filled pore space (WFPS) was calculated from the values of gravimetric soil water content and soil bulk density. Annual N_2_O emissions from the apple orchard soil were estimated from an area after weighing the emissions from three sub-sites (D 0.5, D 1.5 and D 2.5). Annual and seasonal cumulative N_2_O emissions were calculated by integrating weekly fluxes, assuming a constant flux rate between two gas sampling times.
